# Podocyte infolding glomerulopathy after 25 years of clinical remission of lupus nephritis in a patient with systemic lupus erythematosus: A case report and review of literature

**DOI:** 10.1002/ccr3.6756

**Published:** 2022-12-20

**Authors:** Mohamad Hijazi, Tarek Aboursheid, Mohammad Al Termanini, Izzat A. M. Khanjar

**Affiliations:** ^1^ Department of Internal Medicine Hamad Medical Corporation Doha Qatar; ^2^ Rheumatology Department Hamad Medical Corporation Doha Qatar

**Keywords:** lupus nephritis, podocyte infolding glomerulopathy, systemic lupus erythematosus

## Abstract

Podocyte infolding glomerulopathy (PIG) is a rare pathological finding that has gained more recognition recently. Most of the reported cases have been associated with connective tissue diseases especially systemic lupus erythematosus (SLE). Here we report the first case of Infolding Glomerulopathy associated with SLE in the Middle East.

## Abstract 2

Podocyte infolding glomerulopathy (PIG) is a type of kidney injury that might be missed due to lack of available data and unfamiliarity with the disease. Having more cases of PIG reported, especially when successfully treated, will help understanding the pathophysiology of the disease, its clinical manifestations, and the prognosis with varied implemented treatment.

## 1 INTRODUCTION

Podocyte infolding glomerulopathy (PIG) is a rare pathological finding that has gained more recognition in recent years. Most of the reported cases have been associated with connective tissue diseases especially with systemic lupus erythematosus (SLE).

Kidney involvement occurs in almost 30%–70% of lupus patients and is categorized into five subtypes.^1^ However, Infolding Glomerulopathy is not included in this classification. Most of the reported cases of PIG come from Asia.^2^ Here, we report the first case of infolding glomerulopathy associated with SLE in the Middle East, which occurred after a prolonged period of clinical remission of lupus nephritis (LN).

## 2 CASE PRESENTATION

A 52‐year‐old woman was referred to the rheumatology clinic due to bilateral pedal edema with frothy urine. The patient was diagnosed 25 years ago with SLE with LN Class IV. She was treated with prolonged course of prednisolone 60 mg daily with subsequent tapering doses and mycophenolate mofetil (MMF) 1 g twice daily until remission was achieved and maintained. The patient had also history of hypertension, osteoporosis, intracranial benign hypertension, and bilateral hip replacement due to osteonecrosis. On presentation, patient was found to have proteinuria with low complement C3 level. There was no associated arthralgia, skin lesions, or arthritis. The patient denied any shortness of breath or abdominal distension. The review of other systems was unremarkable. Physical examination showed a temperature of 36.2°C, respiratory rate of 16 per minute, blood pressure of 167/90 mm Hg, heart rate of 62 beats per minute, and normal oxygen saturation on room air. There was bilateral pedal edema. However, chest, cardiac, and abdominal examinations were all normal.

Laboratory investigations at presentation revealed 4.28 g protein in 24 h urine collection and normal serum creatinine (Cr). Urine microscopy exam showed 3–5 erythrocytes and 10–12 leukocytes per high power field. Serum C3 was 0.83 g/L and C4 0.19 g/L. Anti‐dsDNA antibody was positive with a titer of (1/40).

A renal biopsy was performed, which showed no morphologic evidence of residual LN. Moderate multifocal tubular atrophy, interstitial fibrosis and mild focal chronic interstitial inflammation were noted. There was also a moderate arteriolosclerosis consistent with history of hypertension.

**FIGURE 1:**
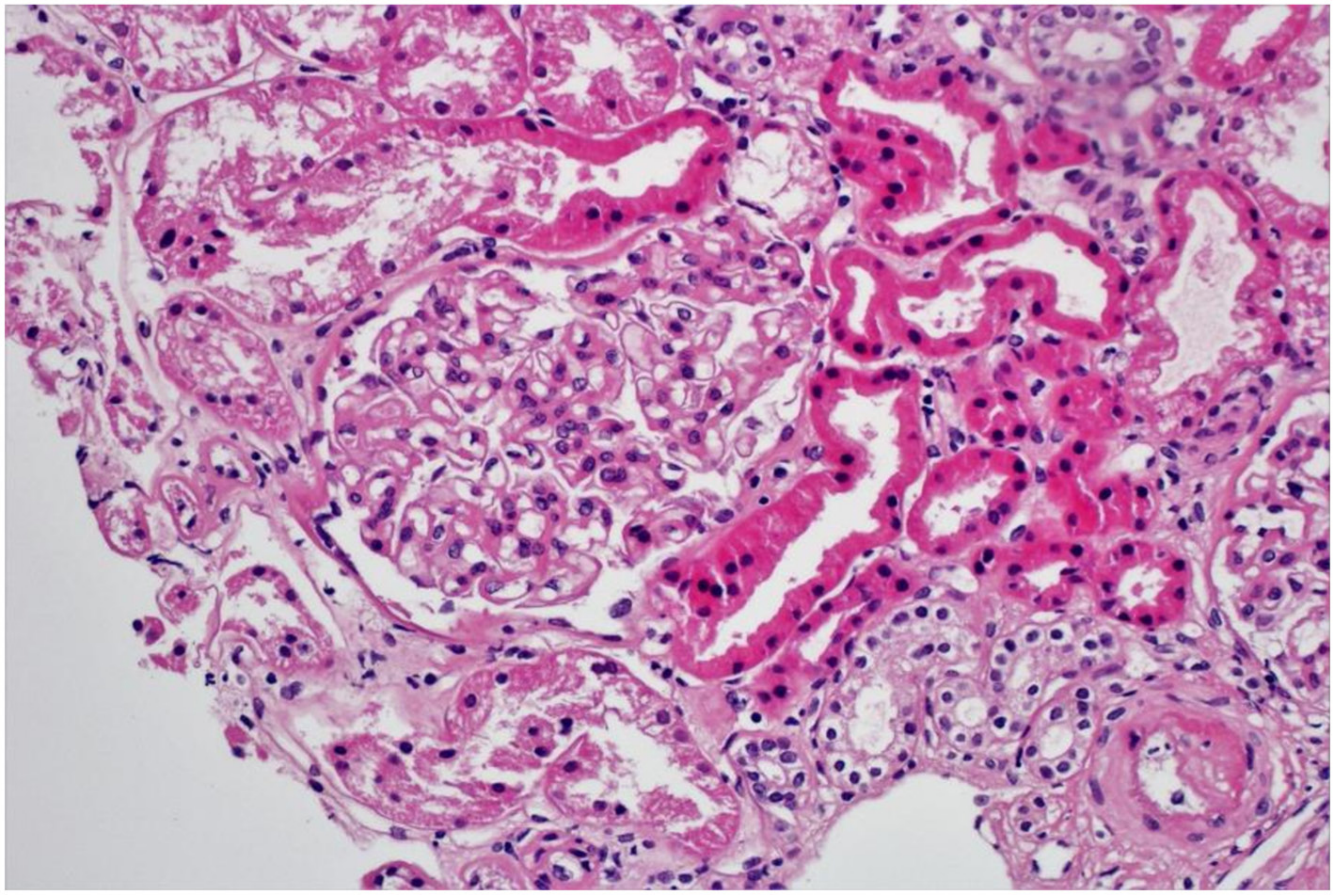
Podocyte infolding glomerulopathy. Light microscopic examination of kidney biopsy shows no definite evidence of lupus nephritis. Only mild and focal mesangial expansion was seen.

Immunofluorescent microscopy was also negative except for +1 IgM present along with the glomerular capillaries. Immunostaining for C4 d also revealed diffuse positivity along the capillary basement membranes (Figure 1).

**FIGURE 2:**
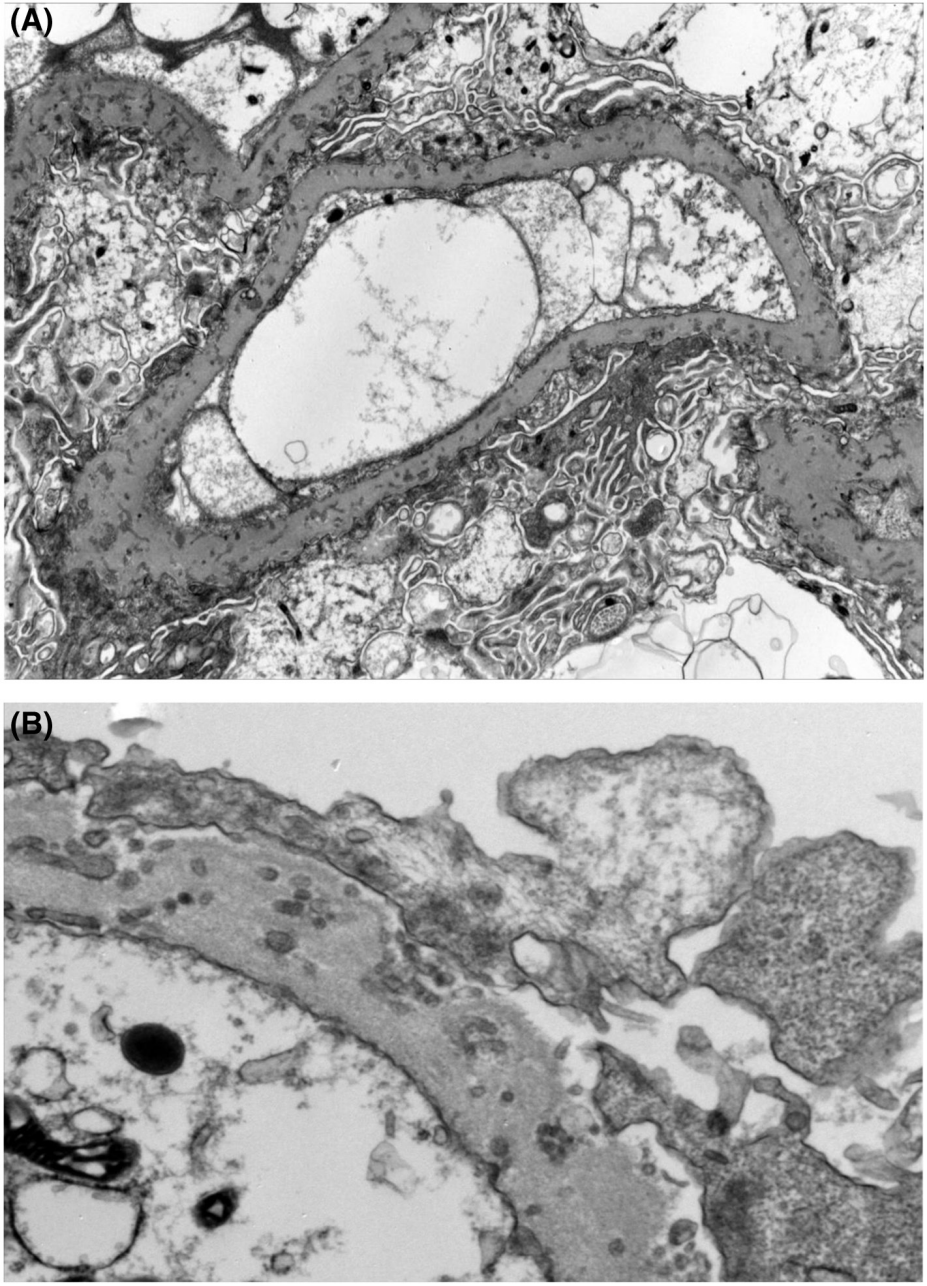
(A) Electron micrograph revealing a glomerular capillary with irregularly thickened basement membrane with embedded deposits composed of cytoplasmic extensions from the foot processes. In addition, there is diffuse effacement of the foot processes. (B) Higher magnification electron micrograph featuring thickened basement membrane and embedded aggregates of spherical structures derived from the foot processes.

A further evaluation of the glomerular changes was done by electron microscopy, which demonstrated widespread subepithelial micro spherular membranous clusters “apparently derived from extensions of the foot processes into the thickened glomerular capillary basement membranes”, and diffuse podocyte foot process effacement. These findings were consistent with a diagnosis of PIG (Figure 2A,B).

Given the confirmed pathologic diagnosis of PIG and the patient's history of hip osteonecrosis and osteoporosis, the decision was made to start the patient on MMF 500 mg twice daily as an alternative to steroids, with regular follow‐up appointments and titration of MMF dose till reaching a maintenance dose of 1 g twice daily. Follow‐up at 3 and 6 months showed that the patient had significant improvement in proteinuria (Urine 24 h protein 0.9 and 0.47 g, respectively) and Anti‐dsDNA Titers (1/19), with resolution of the lower limbs edema (Table 1).

**TABLE 1:** Shows markers of disease activity and reponse to treatment

Laboratory investigations	Hemoglobin (g/dl)	Creatinine (mg/dl)	24 h urine protein (g)	C3/C4 (g/L)	Anti‐dsDNA
Before PIG onset	10	0.8	0.19	0.98/0.16	1/30
During active PIG disease	10.3	0.79	4.28	0.85/0.19	1/40
3 Months after starting treatment	10.1	0.77	0.9	0.99/0.32	1/19

## 3 DISCUSSION

Podocyte infolding glomerulopathy is a rare pathological finding that was first introduced in 2008 in a literature review from Japan^1^ but reports from other regions around the world started to emerge.^2^ Using electron microscopy examination, PIG has been divided into three classes based on the prevalent changes in the glomerular basement membrane (GBM). Class A features primarily podocyte infolding only; class B includes microstructures in the GBM plus primary podocyte infolding, while class C is limited to the presence of only microstructures in the GBM^2^. Renal involvement is known to occur in about 30%–70% of patients with SLE, and almost 10% of the affected patients progress to end‐stage renal disease after 5 years of the initial diagnosis.^3^

Kidney disease in SLE is classically categorized into five classes based on the microscopic finding. However, PIG—though not under this classification—has been reported in association with SLE.

Patients with PIG usually present with proteinuria ranging from 0.3 to 16.8 g/day.^4^ Nephrotic range proteinuria was reported in one‐third of the cases. ^4^ Our patient had a unique clinical course as she was initially diagnosed with class IV LN and was in complete remission. However, after 25 years she presented with proteinuria and low complement level that warranted renal biopsy to exclude relapse or a progression to a different class of LN. It distinctively showed PIG.

Upon literature review, we found 15 cases of PIG in association with SLE apart from our case (Table 1). Thirteen cases from Japan were highlighted in a case review by Joe et al.^2^ The other cases were by Malvar et al.^2^ from Latin America and Zhang et al.^4^ from China.

Out of the 16 cases, 13 were females. The age range was between 23 and 61 years. 24 h Urine protein level was documented in all of these cases, except in Malvar et al.,^2^ and was between 0.5 and 16.8 g with a median value of 1.75 g.

Light microscopy examination showed class V and class II LN in 7 and 4 case, respectively, either at the time of PIG diagnosis or on previous kidney biopsies. Electronic microscopy examination was positive for podocyte infolding in all cases except of Marval et al.^2^ and in our case where a profuse podocyte effacement was evident. The most common finding under the electron microscope was microspheres (15 of 16 cases). Cluster formation was seen only in five biopsies. Microtubules and dense deposits in GBM were noticed in 10 and 5 cases, respectively (Table 2).

**TABLE 2:** Summarize light and electron microscope findings of Nephritis due to PIG

Case no.	Light microscopy	Podocyte infolding	Microsphere	Cluster formation	Microtubules	Dense deposits in GBM	Staining
1	LN class V	Present	Present	Present	Present	Absent	Negative
2	LN class V	Present	Present	Present	Present	Absent	Negative
3	LN class V	Present	Present	Present	Present	Present	G, A, M, C3, C1q, C5b‐9
4	LN class V	Present	Present	Absent	Present	Absent	Negative
5	LN class V	Present	Present	Present	Present	Present	G
6	LN class V	Present	Present	Absent	Present	Present	G
7	LN class V	Present	Present	Absent	Present	Present	G, M, C1q
8	LN class I	Present	Present	Absent	Absent	Absent	Negative
9	LN class II	Present	Present	Absent	Present	Absent	G, A, C3, C1q
10	LN class II	Present	Present	Absent	Present	Absent	G, A, C3, C1q
11	LN class II	Present	Present	Absent	Absent	Present	G, A, M, C3, C1q, C5b‐9
12	LN class II	Present	Present	Absent	Absent	Absent	G, M, C1q
13	MPGN type 3	Present	Absent	Absent	Present	Absent	G, A
14	MGN + bubbling	Present	Present	Absent	Absent	Absent	M
15	Global sclerosis with segmental sclerosis	Podocyte foot effacement	Present	Absent	Absent	Absent	Negative
16	Mild mesangial sclerosis and hypercellularity	Podocyte foot effacement	Present	Present	Absent	Absent	C4 D

A Steroid‐based regimen was the core treatment in all cases except in our case. Ten patients received only prednisolone, while in three other cases either MMF, Cyclosporine, or Tacrolimus + Hydroxychloroquine was used as an add on therapy to prednisolone. Only two of the reported case failed to mention the treatment regimen. Our patient received only MMF to avoid steroids given her previous history of osteoporosis and bilateral hip osteonecrosis.

The reported follow‐up period varied between the cases (Table 3). While most of the cases showed favorable response, two cases had worsening in proteinuria 5 years after treatment. Our patient, who was treated with MMF, had a decrease in proteinuria after 3 months.

**TABLE 3:** Summarizes demographics, coexisting conditions, disease markers, treatment provided and response to treatment

Case no	Age	Gender	Coexisting condition	Hypertension	Creatinine	Proteinuria	Hematuria	Treatment	Response to treatment
1	29	Female	Hydronephrosis due to lupus cystitis	No	0.7	1.6	No	Prednisolone	3.8 g at 5 years
2	46	Female	Hydronephrosis due to lupus cystitis	No	0.5	0.6	No	Prednisolone	0.7 g at 2 years
3	27	Female	N/A	No	0.4	2.7	No	Prednisolone	0.4 g at 6 months
4	53	Male	Bilateral urethral stone	Yes	0.9	3.1	Yes	Prednisolone	Remission after 8 months
5	23	Female	N/A	No	0.5	1.8	Yes	Prednisolone	Remission after 5 years
6	31	Female	N/A	No	0.9	0.5	Yes	Prednisolone	Remission
7	24	Female	Sjogren syndrome	No	0.6	6	No	prednisolone and Pulse steroids 3d	0.15 g at 9 weeks
8	31	Male	N/A	Yes	1.9	0.5	No	Prednisolone 20 mg	Remission
9	37	Female	N/A	No	1.2	1	No	Prednisolone 20 mg, MMF	0.2 g at 2 months
10	40	Female	N/A	No	0.5	1.5	NO	N/A	N/A
11	30	Female	N/A	NO	0.5	1.6	Yes	prednisolone and Pulse steroids 3d	0.5 g at 1 month
12	61	Female	Takayasu's arteritis	Yes	0.5	1.7	No	Prednisolone 40 mg, Cyclosporin	1.6 g at 7 years
13	49	Male	PBC, Sjogren syndrome, Cystitis	yes	1.1	2.2	No	Prednisolone 40 mg	Remission after 9 years
14	23	Female	N/A	No	0.53	16.8	Yes	Prednisolone, Tacrolimus, HCQ	0.3 g at 6 months
15	38	Female	N/A	N/A	N/A	N/A	N/A	N/A	N/A
16	52	Female	N/A	Yes	0.79	4.28	No	MMF	0.47 g at 6 months

Similar to our case, Sugiyamae et al. and Sato et al. reported two female patients who were previously diagnosed with classic form of LN (class II) and achieved complete remission for 2 and 5 years, respectively. These two patients underwent repeated kidney biopsy due to new‐onset proteinuria, which demonstrated the presence of PIG in both cases.^5^, ^6^ The development of PIG after 25 years of clinical remission of LN added a special singularity to our case.

## 4 CONCLUSION

Podocyte infolding glomerulopathy is a rare kidney pathology associated with Lupus and can present for the first time after the relapse of another classic type of LN. Up to our knowledge, only two patients who were diagnosed with SLE acted in the same manner and developed PIG after 3–5 years of remission from other types of LN. What makes our case unique is the paucity of PIG‐reported cases around the world, its occurrence after 25 years of another type of LN, and the line of treatment she received.

## AUTHOR CONTRIBUTIONS

**Mohamad Hijazi:** Conceptualization; data curation; funding acquisition; writing – review and editing. **Tarek Aboursheid:** Resources; writing – original draft. **Mohammad Al Termanini:** Writing – original draft. **Izzat A. M. Khanjar:** Project administration; supervision.

## FUNDING INFORMATION

The publication of this article was funded by Qatar National Library.

## CONFLICT OF INTEREST

The authors have no conflict of interest to disclose.

## CONSENT

Written informed consent was obtained from the patient to publish this report in accordance with the journal's patient consent policy.

## Data Availability

Data subject to third party restrictions.
